# Cytokines in saliva, serum, and temporomandibular joint synovial fluid in children with juvenile idiopathic arthritis: An explorative cross-sectional study

**DOI:** 10.1186/s12969-025-01118-y

**Published:** 2025-06-17

**Authors:** Paula Frid, Josefine M. Halbig, Per Alstergren, Johanna Rykke Berstad, Lena Cetrelli, Astrid Jullumstrø Feuerherm, Berit Flatø, Annika Rosen, Karen Rosendahl, Marite Rygg, Veronika Rypdal, Nils-Thomas Songstad, Berit Tømmerås, Ellen Nordal, Mohammed Al-Haroni

**Affiliations:** 1 Department of Otorhinolaryngology, Division of Oral and Maxillofacial Surgery, University Hospital North Norway, Tromsø, Norway; 2 Public Dental Service Competence Centre of North Norway, Tromsø, Norway; 3 Department of Clinical Dentistry, UiT The Arctic University of Norway, Tromsø, Norway; 4 Scandinavian Center for Orofacial Neurosciences (SCON), Faculty of Odontology, Malmö University, Malmö, Sweden; 5 Orofacial Pain and Jaw Function, Faculty of Odontology, Malmö University, Malmö, Sweden; 6 Specialized Pain Rehabilitation, Skåne University Hospital, Lund, Sweden; 7 Department of ENT and Oral and Maxillofacial Surgery, Oslo University Hospital, Oslo, Norway; 8 Center for Oral Health Services and Research (TkMidt), Trondheim, Norway; 9 Department of Clinical and Molecular Medicine, Faculty of Medicine and Health Sciences, NTNU - Norwegian University of Science and Technology, Trondheim, Norway; 10 University of Oslo, Oslo, Norway; 11 Department of Rheumatology, Oslo University Hospital, Oslo, Norway; 12 Department of Clinical Dentistry, University of Bergen, Bergen, Norway; 13 Department of Oral and Maxillofacial Surgery, Haukeland University Hospital, Bergen, Norway; 14 Department of Radiology, University Hospital North-Norway, UiT The Arctic University of Norway, Tromsø, Norway; 15 Department of Pediatrics, St. Olavs Hospital, Trondheim University Hospital, Trondheim, Norway; 16 Department of Clinical Medicine, UiT The Arctic University of Norway, Tromsø, Norway; 17 Department of Pediatrics and Adolescence Medicine, University Hospital of North Norway, Tromsø, Norway

**Keywords:** Juvenile idiopathic arthritis, Biomarkers, Cytokines, Children, TMJ arthritis, Saliva, Serum, Synovial fluid

## Abstract

**Background:**

Proinflammatory cytokines are central to disease mechanisms and important therapeutic targets in inflammatory chronic diseases. This exploratory study aimed to compare cytokine concentrations in saliva, serum, and temporomandibular joint (TMJ) synovial fluid in children with juvenile idiopathic arthritis (JIA) and controls.

**Methods:**

In this cross-sectional study, we included consecutive children with JIA and TMJ arthritis, planned for a TMJ corticosteroid injection, and non-JIA controls from three different centers in Norway. Data on demographics, disease activity, presence of TMJ arthritis, and medication were obtained. Samples of unstimulated saliva, serum, and TMJ synovial fluid were collected. The amount of recovered synovial fluid in each sample, collected by the push-and-pull technique, was quantified with the hydroxocobalamin method. Cytokine levels were analyzed using Luminex xMAP technology.

**Results:**

Fifteen patients with JIA and TMJ arthritis (JIA-TMJ) (median age 15.0 (interquartile range (IQR) 11.0–16.0) years) and 34 controls (median age 13.0 (IQR 9.8–15.0) years) were consecutively recruited. Samples of saliva (JIA-TMJ, *n* = 13, and controls, *n* = 28), serum (JIA-TMJ, *n* = 11, and controls, *n* = 16), and TMJ synovial fluid (JIA-TMJ, *n* = 8) were collected. In saliva from JIA-TMJ, we found significantly higher levels of the cytokines IL-1β, IL-4, IL-5, IL-9, IL-10, IL-12, IL-13, IL-17, Eotaxin, FGF basic, GM CSF, PDGF bb, TNF, and RANTES, while IP-10 was found in significantly lower concentration compared to controls. In serum, there were no significant differences in these cytokine concentrations between JIA-TMJ and controls. Three TMJ synovial samples fulfilled the strict sampling criteria and were included in the analysis. The level of detected cytokines in TMJ synovial samples was higher in JIA-TMJ compared to controls, as described in a previous Nordic study.

**Conclusions:**

In this exploratory study, several proinflammatory cytokines were found in higher concentrations in saliva in JIA-TMJ compared to saliva from the controls. No differences were seen in serum between the groups. Some pro- and anti-inflammatory cytokines detected in JIA-TMJ synovial fluid were found in higher concentrations compared to TMJ synovial fluid from healthy adult reference data.

**Supplementary Information:**

The online version contains supplementary material available at 10.1186/s12969-025-01118-y.

## Introduction

Juvenile idiopathic arthritis (JIA) is the most common chronic rheumatic disease in children, with a prevalence of 1–2 per 1000 children [1, 2]. The temporomandibular joint (TMJ) is involved in 40–90% [3–5] of children with JIA, and TMJ arthritis can lead to reduced mouth opening, pain, growth disturbances with dentofacial deformity [6–8], and reduced quality of life [9]. TMJ arthritis may also lead to micrognathia with anterior open bite and a reduction in posterior airway space and related comorbidities [8, 10–12]. TMJ arthritis is difficult to diagnose and manage because there may be absence of clinical signs and symptoms. Established clinical criteria for the diagnosis of TMJ arthritis in JIA are not yet available [10], even if consensus-based interdisciplinary recommendations for the management of orofacial manifestations in JIA have been published [13, 14]. Moreover, novel magnetic resonance imaging (MRI)- and cone beam computed tomography (CBCT)-based scoring systems for inflammatory and destructive change, have been devised and validated, to guide both diagnosis and management [15, 16].

The pathogenesis of JIA remains unknown. A combination of genetic, immunologic, and environmental factors has been proposed [17]. Cytokines play a central role in many inflammatory diseases, where complex immune responses could shift either towards a protective or a destructive process [18, 19]. Cytokines may be categorized as proinflammatory (e.g. Interleukin-1 beta (IL-1β), IL-2, IL-6, and tumor necrosis factor (TNF)) or anti-inflammatory (e.g. IL-10 and interferon gamma (IFN)-γ) mediators according to their predominant tissue-specific effects [20]. In chronic inflammatory joint disease, the cytokine balance in synovial fluid is altered, with a predominance of proinflammatory cytokines [21]. Increased levels of several proinflammatory molecules have been found in serum and synovial fluid in affected joints in JIA, and the two proinflammatory cytokines TNF and IL-6 are important therapeutic targets in the treatment of JIA [22].

Biologic disease-modifying anti-rheumatic drugs (bDMARDs), with TNF inhibitors (TNFi) mostly used, have dramatically improved the prognosis in JIA [23], but treatment-resistant disease, disease flares, and subclinical inflammation are still a problem for many children [24, 25]. Composite measures of clinical and biochemical markers have been used to describe, quantify and predict disease activity and outcome in JIA [26, 27]. Highly sensitive and responsive biomarkers are therefore sought to monitor disease activity and flare.

Studies comparing salivary biomarkers from children with JIA and TMJ arthritis and controls are few and show variable results [28–30]. Studies describing TMJ synovial fluid in children with JIA are even more rare. Only one study presents levels of pro- and anti-inflammatory cytokines in TMJ synovial fluid from healthy individuals [31].

Investigating the cytokine concentrations in saliva, serum, and synovial fluid in JIA may increase our understanding of the role of these cytokines in the pathogenesis of JIA and bring further hypothesis of possible interventions. To our knowledge, there are no studies describing cytokine levels in both saliva, serum, and TMJ synovial fluid in children with JIA and TMJ arthritis. Therefore, this exploratory study aimed to investigate the cytokine profile in saliva, serum, and TMJ synovial fluid in children with JIA and TMJ arthritis compared to controls.

## Materials and methods

### Study design and patients

The present cross-sectional study is a project based partly on the NorJIA study (Norwegian JIA Study – Imaging, oral health and quality of life in children with JIA), a larger Norwegian prospective multicenter cohort study registered in Clinical Trials.gov (NCT03904459). For the present study, recruitment and inclusion took place between November 2015 and December 2018 at the Department of Pediatrics and Adolescence Medicine, University Hospital North Norway (UNN), Public Dental Service Competence Centre of North Norway (TKNN), Tromso, Haukeland University Hospital Bergen, and Oslo University Hospital, Rikshospitalet, Oslo.

Inclusion criteria, were children who fulfilled the JIA classification criteria defined by the International League of Associations for Rheumatology (ILAR) [32], age at the study visit < 18 years, active arthritis in one or both TMJs, and a written consent to participate. Controls from the general population, were recruited from the NorJIA main project and consisted of children attending a free routine visit to a dentist according to the regular Norwegian community dental care program with a written consent to participate. The controls did not have JIA or other rheumatic diseases. Study participants were included if they had both medical and oral health examinations and at least one of the three biofluids, serum, saliva, TMJ synovial samples, available for biomarker assessment. For both groups, children with diseases such as cancer, hereditary deformities of the face, or skeletal dysplasia, were excluded. Also, children on antibiotics one week prior to sampling, were excluded.

### Data collection

Data collected for the JIA group were demographics, JIA category, duration and onset of JIA, and medication, as well as disease activity and severity based on clinical examinations, assessed by experienced pediatric rheumatologists. The pediatric rheumatologists were calibrated through regular meetings in the study period with clinical variables thoroughly discussed and defined in a common study protocol based on validated measures of disease activity, including definitions based on the Temporomandibular joint Juvenile Arthritis Working group (TMJaw) recommendations [33]. Active joints were defined according to the general definition of arthritis, as swelling within a joint or limitation in the range of joint movement with joint pain or tenderness [34]. TMJ arthritis was defined as “clinical signs of pain on jaw movement, limitation of maximal incisal opening (MIO), limitation of laterotrusive- or protrusive jaw movements, or dentofacial growth disturbances, and MRI findings suggestive of TMJ arthritis (i.e. active inflammation in the TMJ based on increased contrast enhancement, bone marrow edema and/or effusion, and flattening of the glenoid fossa)”.

Patient-reported global assessment of disease impact on overall well-being (PRgloVAS) within the last week on a 0–10 (21 increments) visual analogue scale (VAS), were registered. On this scale, 0 indicates no activity/no pain/no impact, and 10 indicates maximum activity/worst pain/maximal impact. A complete blood cell count, and results of rheumatoid factor (RF), human leukocyte antigen B27 (HLA-B27), the erythrocyte sedimentation rate (ESR), and C-reactive protein (CRP), were registered. The composite juvenile arthritis disease activity score (JADAS10, range from 0 to 40), was calculated as the simple sum of the medical doctor’s global assessment of overall disease activity on a 10 cm VAS (MDgloVAS, range 0–10), PRgloVAS (range 0–10), active joint count (0 to maximum 10 joints), and the ESR (normalized to 0–10) [35, 36]. Inactive disease was defined according to the American College of Rheumatology (ACR) provisional criteria requiring the following: (1) no active joints; (2) no fever, rash, serositis, splenomegaly, or generalized lymphadenopathy attributable to JIA; (3) no active uveitis; (4) normal ESR or CRP or if elevated, not attributable to JIA; (5) MDgloVAS = 0; and 6), and duration of morning stiffness ≤ 15 min [37].

### Saliva collection procedure

Before the oral examination, the participants collected whole, unstimulated saliva for six minutes. They were told not to drink or eat and only take prescribed medication two hours before the saliva sampling. The salivary flow rates were calculated by dividing the collected volume of saliva by the time used, giving milliliter per minute (mL/min). Medications taken the day before and on the same day of sampling were recorded. Each saliva sample was aliquoted and frozen at -80 °C until further analyses.

## Gingival bleeding index and oral hygiene index

After the saliva sampling and following a standardized protocol, four calibrated specialists in pediatric dentistry and oral and maxillofacial surgery (PF, JH, JRB, AR), performed the oral examination. A modified version of the Gingival bleeding index (GBI) [38] and the Simplified Oral Hygiene Index (OHI-S) [39] were used with six index teeth as earlier described [40].

### Temporomandibular joint synovial fluid collection procedure

The skin in the preauricular area was disinfected with 70% ethanol and 5% chlorhexidine before local anesthesia with an auriculotemporal nerve block was applied. The amount of recovered synovial fluid in each sample, collected by the push-and-pull technique, was quantified with the hydroxocobalamin method, as described by Alstergren et al. [41, 42]. A washing solution consisting of 22% hydroxocobalamin (Behepan^®^ 1 mg/ml) in physiological saline (sodium chloride 9 mg/ml) was used. A TMJ injection with 4 ml washing solution through a stop-cock syringe was performed. One milliliter of washing solution was injected slowly, the valve was turned, and available fluid was aspirated back. This procedure was repeated a total of three times for each TMJ leaving the same cannula inside the joint. If aspiration of the washing solution was possible and the resistance in the syringe was minor during injection, then the needle tip was placed within the joint space. The final aspirate with diluted synovial fluid was frozen and stored at 80 °C before further analyses. The synovial fluid concentration of each cytokine was calculated using the following formula:

$$C_{S F}=\frac{C_{A S P}}{1-\left(A b s_{A S p} / A b s_{\text {wash }}\right)}$$

where *C*_SF_ = synovial fluid concentration of a given cytokine, *C*_ASP_ = aspirate concentration of the given cytokine measured by the Luminex assay, Abs_Asp_ = aspirate absorbance, and Abs_wash_ = washing solution absorbance. Samples that fulfilled the sample quality criteria (dilution factor < 0.98, total aspirate > 0.5 ml, no or only minor blood contamination, and no hemolysis in the analysis were used for cytokine analyses [42]. After sampling of synovial fluid from the upper joint compartment, steroids were injected as described earlier [43]. The injection procedure was performed by experienced specialists in oral and maxillofacial surgery at all centers (PF, AR, JRB). The sampling of saliva, serum, and TMJ synovial biofluids were performed on the same day.

### Cytokine quantification in saliva, serum, and TMJ synovial fluid

A total of 30 µL of unstimulated saliva (1:2 dilution), 20 µL serum (1:3 dilution), and 30 µL TMJ synovial fluid (1:2 dilution) were used for cytokine analysis. Cytokine levels in the samples were analyzed using a multiplex fluorescent bead-based immunoassay. The Bio-Plex Pro Human Cytokine 27-plex Assay Group 1 (Bio-Rad Laboratories, Hercules, CA) identified 27 proteins (the pro-inflammatory cytokines Tumor necrosis factor (TNF), Interleukin (IL)-1β, IL-2, IL-6, IL-7, IL-9, IL-12 (p70), IL-15, IL-17 A; the colony-stimulating factors G-CSF and GM-CSF; the chemokines Eotaxin (CCL11), IL-8, Interferon (IFN)-γ, Interferon Gamma-induced Protein 10 (IP-10), Monocyte Chemoattractant Protein MCP-1 (MCAF), Macrophage Inflammatory Protein-1α (MIP-1α), MIP-1β, Regulated on Activation, Normal T Cell Expressed and Secreted (RANTES); and the anti-inflammatory cytokines IL-1Ra, IL-4, IL-5, IL-10, IL-13, as well as growth factors, such as Platelet-derived growth factor-BB (PDGF-BB), Fibroblast Growth Factor-basic (FGF-basic), and Vascular endothelial growth factor (VEGF) with a workflow as described by Houser [44]. The Bio-Plex^®^ 200 Systems (Bio-Rad Laboratories, Hercules, CA) were used, including the microplate platform and Bio-Plex Manager™ 6 software. The analysis was done according to the manufacturer’s recommendations based on the Luminex xMAP technology. The amount of protein in each sample was extrapolated from standard curve ranges with concentrations reported in pg/ml. Cytokine concentrations (pg/ml) within the standard curve range were used. Values below the limit of detection (LOD) were set to “Lower level of quantitation/2” (LLOQ/2) [45]. Cytokines were excluded if more than 60% of the samples were below LOD.

### Statistical and bioinformatic analyses

Descriptive statistics were used to present clinical and demographic data, with median (interquartile range, IQR), mean (standard deviation, SD), and frequencies. Non-parametric statistics were used for the overall non-normally distributed cytokine data. Associations between patients with JIA-TMJ and controls and between JIA-TMJ and different disease characteristics were analyzed using chi-square or Fisher´s exact test for categorical variables, as appropriate. The Student´s t-test was used for continuous variables if reasonably normally distributed; otherwise, the Mann-Whitney U-test for two independent samples was used. A P-value ≤ 0.05 was considered statistically significant for clinical characteristics. Statistical analysis was performed using SPSS software, version 29.

## Results

### Participants

Fifteen children with newly diagnosed TMJ arthritis were recruited (Tromsø *n* = 12, Bergen *n* = 1, Oslo *n* = 2), together with 34 controls from Tromsø. Saliva samples (*n* = 13 JIA-TMJ, *n* = 28 controls), serum samples (*n* = 11 JIA-TMJ, *n* = 16 controls), and TMJ synovial fluid samples (*n* = 3) were analyzed.

### Demographics and disease characteristics

The demographics and oral characteristics of the study groups are reported in Table 1. There was a female predominance in both groups, and the median age at examination was 15 years for the JIA-TMJ group and 13 years for the controls. There was no significant difference in salivary flow rate between children with JIA-TMJ and controls. The Gingival bleeding Index (GBI) and the Simplified Oral Hygiene Index (OHI-S) were, however, significantly higher in children with JIA and TMJ-arthritis.

**Table 1:** Characteristics among children with juvenile idiopathic arthritis and temporomandibular joint arthritis and controls

Characteristics	JIA-TMJ (*n* = 15)	Controls (*n* = 34)	Cut-off	*P*-value*
**Demographics**				
Female, no. (%)	12 (80)	27 (79)		0.642^a^
Age at sampling, years	15 (11–16)	13 (10–15)		0.063 ^c^
Age at disease onset	11 (8–14)			
**Centers**, no. (%)				
Tromsø	12 (80)	34 (100)		
Oslo	2 (13)			
Bergen	1 (7)			
**Oral characteristics**				
Bleeding index, %	44 (0–53) (*n* = 9)	6 (0–11) (*n* = 25)	> 10	0.001^b^
The Simplified Oral hygiene index (OHI-S)	0.7 (0.4–1.1) (*n* = 9)	0.3 (0.0-0.4) (*n* = 25)		0.011^c^
Saliva flow rate unstimulated (ml/min)	0.3 (0.1–0.3) (*n* = 11)	0.4 (0.2–0.7)	≤ 0.1	0.177 ^c^

Values are the median (1st to 3rd quartile) unless indicated otherwise. Chi-square test (Fischer’s exact test)^a^ is used for testing associations between groups. When testing two independent groups (continuous variables); Student T-test^b^ and Mann-Whitney U test^c^; *P < 0.05 for statistical significance; JIA, juvenile idiopathic arthritis; TMJ, temporomandibular joint

Disease characteristics in the JIA-TMJ group are reported in Table 2. Persistent oligoarthritis was the most common category in the JIA-TMJ group (40%). Nine out of 15 patients were either on methotrexate and/or bDMARDs.

**Table 2:** Disease characteristics among children with JIA and TMJ-arthritis (*n* = 15)

	JIA-TMJ (*n* = 15)
**Disease duration**,** years**	1 (0–5)
JIA category*,** n (%)**	
Persistent oligoarthritis	6
Extended oligoarthritis	2
Polyarthrtitis RF neg	3
Psoriatic arthritis	1
Enthesitis related arthritis	2
Undifferentiated arthritis	1
**Disease activity variables**	
Patients with active disease**, n (%)	15 (100)
Patients with active joints, n (%)	15 (100)
Number of active joints	1 (1–2)
MD global VAS	5 (2–7) (*n* = 11)
HLA-B27, no (%)	3 (27) (*n* = 11)
RF, no (%)	0 (0) (*n* = 10)
JADAS10	14 (12–31) (*n* = 10)
**Type of Medication**	
No DMARDs, n (%) ***	6 (40)
MTX only, n (%)	3 (20)
bDMARDs, n (%) ****	6 (40)

Values are median (1st to 3rd quartile) unless indicated otherwise

JIA, juvenile idiopathic arthritis; TMJ, temporomandibular joint; MD global VAS, Medical doctor global assessment of overall disease activity on Visual Analogue Scale (range 0–10); HLA-B27, human leukocyte antigen B27; RF, Rheumatoid factor; JADAS10, the Juvenile Arthritis Disease Activity Score based on 10 joints (range 0–40); DMARDs, Disease-Modifying Anti-Rheumatic Drugs; MTX, methotrexate; bDMARDs, biologic DMARDs

*According to The International League of Associations for Rheumatology (ILAR) classification criteria for JIA

** Active disease / TMJ arthritis was defined as clinical signs and symptoms in addition to signs of arthritis on magnetic resonance imaging (MRI)

*** no systemic medication, ongoing

**** biologics only or in combination with methotrexate, ongoing

### Cytokine analysis

**Fig. 1:**
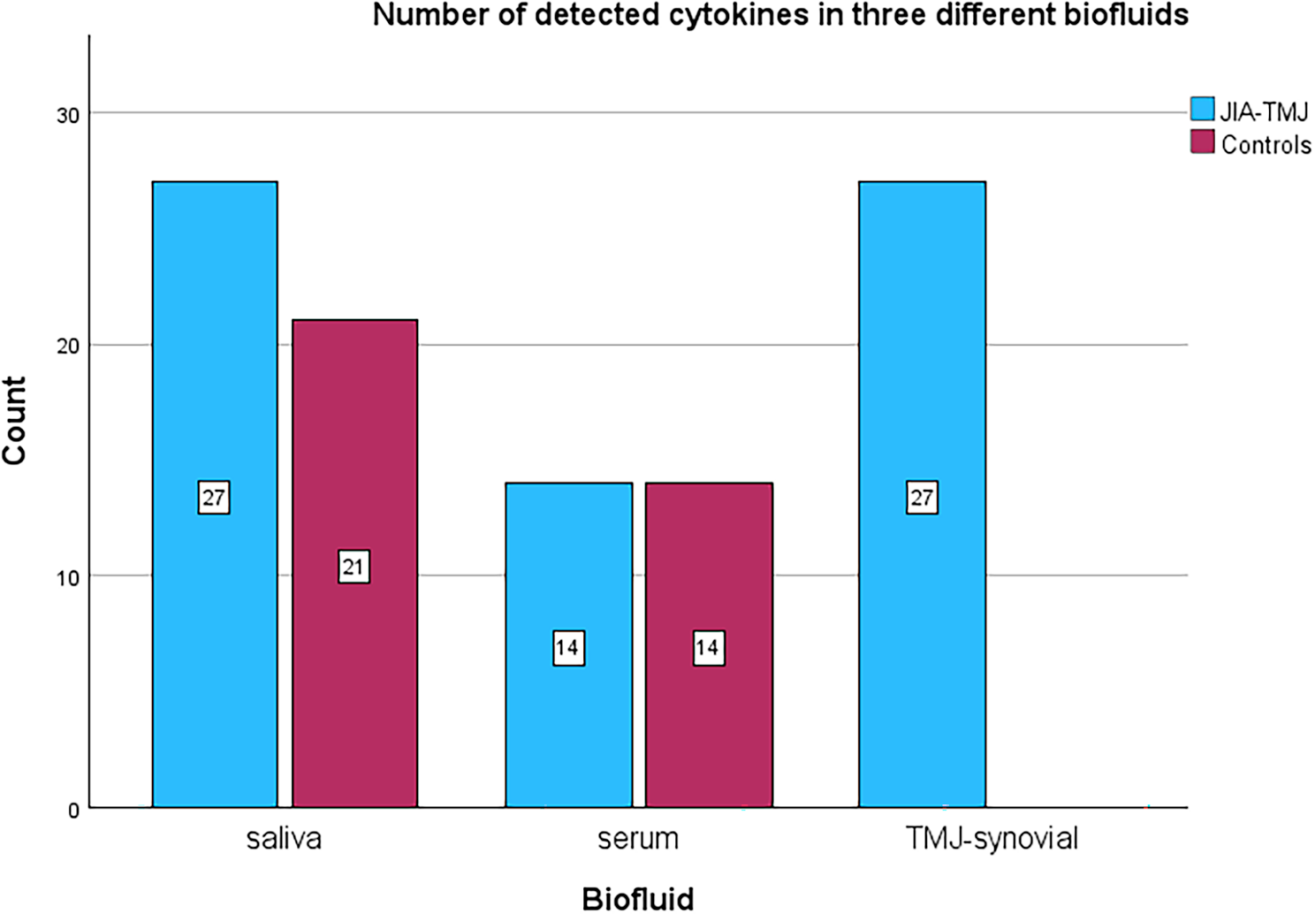
Number of detected cytokines in three different biofluids in children with JIA and TMJ arthritis and in non-JIA controls. Thirteen cytokines were common for all groups and biofluid: IL-4, IL-8, IL-9, IL-13, IL-17, Eotaxin, GCSF, IP10, MCP1MCAF, MIP1a, PDGFbb, RANTES, TNF

Thirteen cytokines were common for all groups and biofluids (Supplemental Table 1, Fig. 1).

***Cytokine concentrations in unstimulated saliva*** in children with JIA-TMJ (*n* = 15) and controls (*n* = 34) are presented in Table 3. Fourteen of the detected 27 cytokines in saliva were significantly higher in JIA-TMJ compared to controls (proinflammatory and anti-inflammatory). The proinflammatory cytokine IP-10 (CXCL10) was significantly lower in JIA-TMJ saliva compared to controls (Table 3).

**Table 3:** Cytokine concentrations in saliva among children with JIA and TMJ arthritis and controls

Cytokines	JIA-TMJ (*n* = 13) (pg/ml), median (IQR)	Controls (*n* = 28) (pg/ml), median (IQR)	*P*-value
**IL-1b**	43.6 (21.0-116.1)	21.9 (9.3–46.4)	0.05 ^b^
IL-1ra	23463.0 (12203.6- 42211.4)	15201.2 (10366.4- 27364.0)	0.313 ^b^
IL-2	5.2 (2.5–8.5)		
**IL-4**	2.4 (1.9–3.4)	1.6 (1.2–2.5)	0.015 ^b^
**IL-5**	57.4 (10.4–83.9)	26.4 (5.9–43.5)	0.030 ^b^
IL-6	3.9 (1.9–5.8)	2.7 (1.9–5.1)	0.465 ^b^
IL-7	5.9 (5.9–25.5)	5.9 (5.9–5.9)	0.056 ^b^
IL-8	334.7 (139.7-794.3)	398.8 (202.6-643.7)	0.933 ^b^
**IL-9**	25.1 (17.2–36.2)	15.2 (11.3–20.5)	0.004^a^
**IL-10**	5.1 (2.7–7.9)	0.6 (0.6–3.9)	0.001 ^b^
**IL-12**	22.1 (7.1–25.8)	0.8 (0.8–6.6)	< 0.001 ^b^
**IL-13**	1.2 (0.9–2.3)	0.7 (0.5–1.2)	0.004 ^b^
IL-15	39.1 (39.1-174.8)		
**IL-17**	13.7 (10.2–21.9)	7.4 (1.4–11.6)	< 0.001 ^b^
**Eotaxin**	2.6 (2.0-3.4)	2.0 (1.7–2.5)	0.009 ^a^
**FGF basic**	19.1 (14.9–26.4)	15.2 (12.3–18.4)	0.018 ^b^
G CSF	126.3 (82.0-173.7)	100.0 (70.1-164.7)	0.566 ^b^
**GM CSF**	3.7 (2.5–6.6)	2.3 (1.9–3.9)	0.025 ^b^
IFNg	206.9 (176.4-232.7)	188.1 (163.4-215.9)	0.645 ^a^
**IP10**	28.1 (16.7 -101.4)	123.1 (65.6-240.7)	0.011 ^b^
MCP1 MCAF	25.7(14.7–51.9)	18.4 (14.5–48.9)	0.674 ^b^
MIP1a	1.1 (0.7–1.9)	0.9 (0.7–1.3)	0.457 ^b^
**PDGFbb**	38.2 (24.3–48.0)	13.7 (1.8–32.3)	0.008 ^b^
MIP1b	3.6 (0.9–12.9)		
**RANTES**	22.4 (16.7–25.7)	16.9 (13.5–21.4)	0.048 ^a^
**TNF**	29.5 (21.9–68.7)	22.7 (15.7–30.9)	0.039
VEGF	144.3 (15.0-266.5)		

Values below the limit of detection were set to “Lower level of quantitation/2” (LLOQ/2). **Cytokines in bold are significantly different between the groups.** The following cytokines were not reported in controls due to more than 60% of the samples were below the limit of detection: IL-2, IL-15, MIP1b, and VEGF. When testing two independent groups (continuous variables); ^a^Student T-test if normally distributed, and ^b^Mann-Whitney U test if not normally distributed; *P* < 0.05 for statistical significance. One of the patients had three saliva samples (same time point) and therefore the mean was calculated from those samples

***Cytokine concentrations in serum*** showed no significant differences between JIA-TMJ and controls (Table 4). Fourteen cytokines were detected in both groups. There was no significant difference in serum TNF concentration between users of TNF inhibitors (TNFi) compared to those not using TNFi (results not shown).

**Table 4:** Cytokine concentration in serum among children with JIA and TMJ arthritis and controls

Cytokines	JIA-TMJ (*n* = 11) (pg/ml), median (IQR)	Controls (*n* = 16) (pg/ml), median (IQR)	*P*-value
IL-4	1.9 (1.4–3.2)	1.6 (1.1–2.1)	0.113^a^
IL-8	3.4 (0.5–5.5)	2.9 (0.5-4.0)	0.216^b^
IL-9	330.9 (318.7-352.1)	320.3 (307.6-340.9)	0.580^a^
IL-13	0.9 (0.1–1.1)	0.1 (0.1–1.7)	0.126^b^
IL-17	18.4 (16.4–24.7)	17.7 (16.6–19.2)	0.371^b^
Eotaxin	38.8 (33.1–58.2)	37.3 (30.2–46.1)	0.348^b^
G CSF	84.0 (62.3-113.4)	66.7 (62.3–92.5)	0.189^b^
IP10	182.4 (76.0-315.9)	177.6 (133.6-223.7)	0.844^b^
MCP1 MCAF	12.4 (9.1–22.4)	10.6 (7.2–16.6)	0.235^b^
MIP1a	0.9 (0.6–1.3)	0.6 (0.4-1.0)	0.681^a^
PDGFbb	1340.2 (1090.6–2101.0)	1249.5 (794.3-1565.4)	0.681^a^
MIP1b	110.3 (102.6-114.7)	108.7 (103.5-113.7)	0.961^b^
RANTES	7108.5 (6325.6-7482.6)	6375.3 (5984.6-6733.6)	0.585^a^
TNFa	144.7 (131.3-150.8)	144.7 (129.4-148.3)	0.334^a^

Values below the limit of detection were set to “Lower level of quantitation/2” (LLOQ/2). The following cytokines were not reported in JIA-TMJ serum and controls-serum due to more than 60% of the samples were below the limit of detection: IL-1ra, IL-1b, IL-2, IL-5, IL-6, IL-7, IL-10, IL-12, IL-15, GM-CSF, IFN γ, FGF-basic, VEGF. When testing two independent groups (continuous variables); ^a^Student T-test if normally distributed and ^b^Mann-Whitney U test if not normally distributed; *P* < 0.05 for statistical significance. One of the patients had two serum samples and therefore the mean was calculated from those samples

***Cytokine concentrations in TMJ synovial fluid.*** Eight of 15 children (9 TMJs) with TMJ arthritis went through the push-pull technique. Only 3 of the 9 TMJ synovial samples fulfilled the sample quality criteria [42]. The reasons for not fulfilling the sample criteria in six samples were blood contamination, not measuring the absorbance in aspirate and washing solution, and a dilution factor above 0.98. The distribution of cytokine concentrations in TMJ synovial fluid are presented in Supplemental Table 1. Among the 27 detected cytokines in TMJ synovial fluid, VEGF and IL-15 were in higher concentrations compared to saliva in JIA-TMJ. IL-5 was in higher concentration in TMJ synovial fluid compared to saliva in both JIA-TMJ and controls.

***The distribution of cytokine concentrations in the three biofluids*** in the two patients who had both saliva, serum, and TMJ synovial fluid samples, are given in Table 5. Although there were differences between the two patients in cytokine concentrations, similar patterns of concentrations were found especially in the following cytokines: IL-9, IP-10, MCP-1, PDGF, and TNF, where concentrations in TMJ synovial fluid were comparable to the serum values.

**Table 5:** Cytokine concentrations in *two* children with JIA and TMJ arthritis having all three biofluids

	Saliva JIA-TMJ		Serum JIA-TMJ		TMJ-Synovial fluid	
Cytokines	Patient 1 (pg/ml)	Patient 2* (pg/ml)	Patient 1 (pg/ml)	(pg/ml) Patient 2* (pg/ml)	Patient 1 (pg/ml)	Patient 2 (pg/ml)
	385.7	90.8	0.2	0.2	0.4	8.0
IL-1ra	74861.2	29156.3	18.2	18.2	41.4	6400
IL-2	12.3	7.5	0.6	0.6	1.4	31.5
IL-4	5.8	3.1	1.9	1.9	0.3	7.5
IL-5	122.3	86.5	2.9	2.9	96.3	2270.0
IL-6	6.1	4.8	0.2	0.2	0.4	38.5
IL-7	30.2	25.3	5.9	5.9	13.5	296.8
IL-8	1712.5	315.2	4.5	0.5	5.2	23.0
IL-9	39.5	32.9	318.7	329.7	308.5	253.0
IL-10	8.3	9.3	0.6	0.6	1.3	132.5
IL-12	25.8	33.7	0.8	0.8	33.3	256.0
IL-13	2.0	2.6	0.7	0.9	2.1	23.0
IL-15	260.2	166.0	39.1	39.1	88.9	1956.5
IL-17	37.9	19.2	16.4	20.8	15.9	71.5
Eotaxin	3.9	3.6	35.7	38.8	27.8	13.5
FGF basic	37.8	22.9	2.1	2.1	4.7	104.0
G CSF	126.3	82.9	96.7	71.1	45.5	177.5
GM CSF	9.4	7.0	0.2	0.2	8.4	48.5
IFNγ	231.2	239.3	0.6	2.3	20.3	153.5
IP10	17.6	15.8	260.9	115.6	39.9	40.0
MCP1 MCAF	58.1	45.7	9.8	9.3	15.5	13.5
MIP1a	1.5	0.8	1.3	0.7	0.6	2.0
PDGFbb	81.2	38.2	578.3	1090.6	397.2	890.0
MIP1b	22.6	4.8	110.5	104.3	112.6	43.5
RANTES	31.4	26.0	5839.3	6325.6	2158.0	100.0
TNF	68.7	59.0	145.7	144.7	149.7	94.0
VEGF	267.4	212.1	3.1	3.1	486.3	8536.5

*One of the patients had two serum and three saliva samples and therefore the mean was calculated from those samples

Cytokine concentrations in TMJ synovial fluid compared to median values of cytokine concentrations in available saliva and serum among all children with JIA and TMJ arthritis are presented in Table 6. IL-8, IL-13, and MCP1MCAF were significantly higher in saliva compared to serum in JIA-TMJ while IL-9, Eotaxin (CCL11), IP-10, PDGF-BB, MIP1b, RANTES, and TNF were lower in saliva (Table 6).

**Table 6:** Cytokine concentrations in biofluids among children with JIA and TMJ arthritis

Cytokines	Saliva JIA-TMJ (*n* = 13) (pg/ml) median (IQR)	Serum JIA-TMJ (*n* = 11) (pg/ml) median (IQR)	*P*-value	TMJ-Synovial fluid		
				Patient 1 (pg/ml)	Patient 2 (pg/ml)	Patient 3 (pg/ml)
IL-1b	43.6 (21.0-116.1)			0.4	8.0	0.7
IL-1ra	23463.0 (12203.6- 42211.4)			41.4	6400	82.7
IL-2	5.2 (2.5–8.5)			1.4	31.5	2.9
IL-4	2.4 (1.9–3.4)	1.9 (1.4–3.2)	0.213^b^	0.3	7.5	0.7
IL-5	57.4 (10.4–83.9)			96.3	2270.0	116.6
IL-6	3.9 (1.9–5.8)			0.4	38.5	0.9
IL-7	5.9 (5.9–25.5)			13.5	296.8	27.0
**IL-8**	334.7 (139.7-794.3)	3.4 (0.5–5.5)	0.001 ^b^	5.2	23.0	2.1
**IL-9**	25.1 (17.2–36.2)	330.9 (318.7-352.1)	0.001 ^b^	308.5	253.0	237.1
IL-10	5.1 (2.7–7.9)			1.3	132.5	12.1
IL-12	22.1 (7.1–25.8)			33.3	256.0	49.4
**IL-13**	1.2 (0.9–2.3)	0.9 (0.1–1.1)	0.05 ^b^	2.1	23.0	3.2
IL-15	39.1 (39.1-174.8)			88.9	1956.5	177.9
IL-17	13.7 (10.2–21.9)	18.4 (16.4–24.7)	0.107 ^b^	15.9	71.5	6.5
**Eotaxin**	2.6 (2.0-3.4)	38.8 (33.1–58.2)	0.001 ^b^	27.8	13.5	25.2
FGF basic	19.1 (14.9–26.4)			4.7	104.0	9.5
G CSF	126.3 (82.0-173.7)	84.0 (62.3-113.4	0.173 ^b^	45.5	177.5	16.1
GM CSF	3.7 (2.5–6.6)			8.4	48.5	8.9
IFNγ	206.9 (176.4-232.7)			20.3	153.5	2.6
**IP10**	28.1 (16.7 -101.4)	182.4 (76.0-315.9)	0.016 ^b^	39.9	40.0	127.5
**MCP1 MCAF**	25.7(14.7–51.9)	12.4 (9.1–22.4)	0.022 ^b^	15.5	13.5	23.1
MIP1a	1.1 (0.7–1.9)	0.9 (0.6–1.3)	0.271 ^b^	0.6	2.0	0.2
**PDGFbb**	38.2 (24.3–48.0)	1340.2 (1090.6–2101.0)	0.001 ^b^	397.2	890.0	55.9
**MIP1b**	3.6 (0.9–12.9)	110.3 (102.6-114.7)	0.001 ^b^	112.6	43.5	74.6
**RANTES**	22.4 (16.7–25.7)	7108.5 (6325.6-7482.6)	0.001 ^b^	2158.0	100.0	1068.4
**TNF**	29.5 (21.9–68.7)	144.7 (131.3-150.8)	0.001 ^b^	149.7	94.0	99.6
VEGF	144.3 (15.0-266.5)			486.3	8536.5	607.2

Significant cytokine differences in bold. When testing two independent groups (continuous variables); Student t-test^a^ and Man Whitney U test^b^. *P* < 0.05 for statistical significance. The following cytokines were excluded in JIA-TMJ serum due to more than 60% of the samples were below limit of detection: IL-1ra, IL-1b, IL-2, IL-5, IL-6, IL-7, IL-10, IL-12, IL-15, GM-CSF, IFNγ, FGF-basic, VEGF

## Discussion

To our knowledge, this is the first study investigating levels of inflammatory cytokines in TMJ synovial fluid, serum, and unstimulated saliva, in children with JIA and TMJ arthritis. We also compare serum and salivary cytokine levels from controls without JIA. The results showed a high variability in concentrations of 27 different cytokines in the three biofluids and between groups. Several pro- and anti-inflammatory cytokines were found in higher levels in saliva, while no differences in serum cytokine levels were found between children with JIA and TMJ arthritis and controls.

### Cytokines in saliva

As in our study, increased concentrations of salivary proinflammatory cytokines were found to be associated with oral inflammation in a systematic review [46]. Higher concentrations of salivary IL-1β and IL-10 in our study measured by Luminex technology, are in line with Rinderknecht et al. [47]. The authors found that most salivary cytokines correlated positively with age and the presence of oral pathologies such as gingivitis and caries in their cross-sectional study of healthy children and adolescents aged 4–18 years. Correlation with oral pathologies in the studies by Diesch et al. [46] and Rinderknecht et al. [47] are in line with our results where gingivitis / the GBI-index and the Simplified Oral Hygiene Index (OHI-S) was significantly higher in children with JIA and TMJ arthritis compared to controls. Higher gingival inflammation in patients with JIA compared to healthy controls is in line with some studies [48–52], while others found no significant difference [53–59]. The difference may be due to different study design and measurement techniques of gingival inflammation [60]. We found no difference in frequency of tooth brushing between JIA and healthy controls that could explain the higher GBI and OHI-S. However, we do not know how effective tooth brushing was performed, some of the children with JIA-TMJ may have had restricted wrist, finger or jaw movements affecting the quality of the tooth brushing. Also, pain from gingival inflammation may restrict the quality of tooth brushing, leading to formation of plaque and dental calculus (i.e. higher OHI-S).We know from our previous report including the same patients, studying the salivary oral microbiome of children with JIA [40] that, after adjusting for dental plaque and calculus (OHI-S), JIA was not found to be a predictor for gingival inflammation in terms of higher GBI. However, there was no overlap between overabundant bacteria associated with GBI and those associated with JIA in that study. Overabundance of bacteria associated with chronic inflammation in JIA could therefore be explained by a disruption of microbial hemostasis in JIA leading to a different cytokine response in biofluids in JIA, also leading to gingival inflammation.

Upregulated levels of the proinflammatory cytokines IL-1β and TNF in saliva in JIA-TMJ are in line with other studies analyzing adult saliva in inflammatory diseases [61, 62]. In one study [63], patients with severe periodontitis were found to have higher salivary concentrations of IL-1β and MMP-8. The authors collected stimulated saliva samples and analyzed biomarkers using ELISA, immunofluorometric assay, or Luminex assays.

Cetrelli et al. found most inflammatory biomarkers in higher levels in serum and lower levels in saliva in JIA compared to controls, and in active compared to inactive disease [28]. Common cytokines for both studies included the salivary biomarkers TNF, IL-6, IL-7, IL-10, IL-17, CCL11 (Eotaxin), and VEGF. In contrast to Cetrelli et al., we detected higher levels of these biomarkers in JIA-TMJ saliva. The reasons for these differences may be different cytokine assays, the number of patients with JIA was different (*n* = 15) compared to Cetrelli et al. (*n* = 42), and our study group had higher inflammatory activity in terms of TMJ arthritis and oral gingivitis [47]. However, both studies found higher salivary levels of CCL11 (Eotaxin) in JIA compared to controls. This may be due to oral inflammation [64] and a disruption of microbial hemostasis in JIA / JIA-TMJ leading to a different cytokine response, as mentioned above [40].

Carlsson and coworkers found a higher concentration of IL-6 in parotid saliva in 45 children with JIA and TMJ arthritis compared to plasma [30]. Also in contrast to our study, Collin and coworkers did not find any differences in 14 detectable cytokines between children with JIA and healthy controls when analyzing stimulated whole saliva using a different immunoassay with 21 targeted biomarkers, including some of the same cytokines as we analyzed [29].

We found significantly higher concentrations of salivary GM-CSF in JIA-TMJ compared to controls. This biomarker is a growth factor and an anti-inflammatory cytokine that stimulates the survival, proliferation, and differentiation of mononuclear phagocytes, which has been implicated in several inflammatory diseases. Dikilitas et al. [65] found that GM-CSF expression was increased in periodontitis stage II (SII-P), stage III (SIII-P), and stage V (SIV-P) in 126 individuals. This is in line with our study showing an association between GM-CSF and the group with increased GBI-index.

We also found a higher concentration of salivary anti-inflammatory FGF-Basic which influences chemotaxis, cell differentiation, proliferation, and tissue regeneration in general. This biomarker plays a role in wound healing [66] of cartilage [67] and affects regeneration in periodontal and bone tissues [68, 69]. Again, this may explain why the group with increased GBI-index in our study had higher salivary concentration of FGF-Basic compared to controls.

In line with our study, Relvas et al. [70] found a significant decrease in the levels of chemokine IP-10 in the saliva of patients with stage III/IV periodontitis compared to healthy controls. Additionally, Cetrelli et al. [28] found, lower concentration of the chemokine IP-10 in the saliva of participants with JIA compared to controls, in line with our study.

In another study, the following salivary biomarkers have recently been detected in children with JIA and controls in similar levels, not in line with our study: TNF, TNFRSF1B, MMP-2, MMP-3, IL-1alpha (IL-1α), IL-1β, IL-6Rα, IL-8, S100A8, CCL2, CCL3, IL-10, CCL11, and CXCL9 [29].

### Cytokines in serum

We found no differences in serum biomarker levels between JIA and controls. This is contrary to Cetrelli et al. [28], who detected higher serum levels in JIA and active disease, especially higher levels of TNF and S100A12. Other studies have also reported higher serum levels of TNF in JIA, especially those treated with TNFi [28, 71]. The reasons for these differences may be that in our study we used a different cytokine assay, the number of patients with JIA was different (*n* = 15) compared to Cetrelli et al. (*n* = 42), and our study group was more active with TMJ arthritis and oral gingivitis [47]. Another study detected higher levels of IL-1β, IL-6, and IL-12, cytokines that have been suggested as biomarkers of disease activity in serum in JIA [72]. In line with our study, Spîrchez and coworkers did not find any significant correlation of serum TNF and IL-1α levels with disease activity in JIA. However, they suggested IL-6 to serve as a biomarker of JIA activity and severity [73].

In the prospective study of Walters and coworkers [71], JIA subjects with increased disease activity were found to have higher proinflammatory cytokines in serum. The use of TNFi resulted in reduced IL-6 and IL-8 levels together with clinical improvement but was also associated with elevated TNF and IL-17 [71]. We have no explanation for the fact that we did not see any difference in TNF concentration in serum in the control group nor when comparing TNFi users versus non-TNFi users. This might be due to different analytic methods for detecting TNF-TNFi complexes in different kits or also be a difference by chance due to a small sample size and different patient selection compared to other studies.

### TMJ synovial fluid

In TMJ synovial fluid from young adult healthy volunteers, using the hydroxocobalamin sampling method, Kristensen et al. [31] quantified the proinflammatory cytokines (reported as pg/ml, medians with interquartile ranges) IL-1β (undetectable), IL-2 (1.8 (0–22)), IL-6 (undetectable), and TNF (23 (13–37)), and the anti-inflammatory cytokines IL-10 (undetectable), and IFN-γ (10 (0–47)), to serve as reference values for future studies on TMJ pathologies [31]. In our study, all these cytokines were seen in a higher concentration in TMJ synovial fluid in children with JIA and TMJ arthritis, suggesting that those cytokines are more specific for inflammatory environments. However, no reference data for children exists.

### Comparison of biofluids

Lower concentration of the cytokine IL-1ra in TMJ synovial fluid compared to saliva was found in our study. This cytokine is an effective inhibitor of the proinflammatory effect of IL1β and is probably important to maintain homeostasis [74].

Consistent with our results, Vignola et al. [75] found VEGF concentrations in the synovial fluid in patients with JIA to be significantly higher than in corresponding sera, suggesting that this pro-angiogenic factor may have a role in the local inflammatory response [75].

### Study strengths and limitations

Both saliva, serum, and TMJ synovial samples were directly frozen and aliquoted before further processing, which is a strength regarding the assessment of cytokine profile. The hydroxycobalamin method enabled measurement of the total volume of the synovial fluid recovered, and true synovial fluid cytokine concentrations could be calculated [41, 42]. However, other methods for TMJ fluid sampling are described in the literature, for example by arthrocentesis with no compensation for the joint washing [76–78] or by pure aspiration [79] where the ratio between the measured cytokine concentration and the total protein content of the aspirate is not calculated. Most of our study subjects were females diagnosed with persistent oligoarthritis in accordance with most population-based studies on patients with JIA, pointing to the representability of our study cases. Our results must, however, be evaluated in the context of a low sample size. The sample size of TMJ synovial fluid was very small with only 3 samples, making statistical comparisons unfeasible. Additionally, only two subjects provided matched saliva, serum, and TMJ synovial fluid samples. Also, due to the cross-sectional study design we could not directly evaluate associations over time. The sampling of saliva, serum, and TMJ synovial biofluids were performed at daytime during the same day, which is a strength of the study even though concentrations of cytokines may vary also during the day.

We used a multiplex fluorescent bead-based immunoassay identifying 27 cytokines only. Other assays may identify more or other biomarkers and give additional information [28, 80].

## Conclusion

A higher concentration of several proinflammatory cytokines was found in saliva of children with JIA and TMJ arthritis compared to controls, while other cytokines such as IP-10 was found in lower levels in saliva. No differences in cytokine concentrations were seen in serum between JIA-TMJ and controls. In TMJ synovial fluid, the cytokines TNF, Il-2, and INF-γ were up-regulated together with IL-1β, Il-6, and IL-10 compared to healthy adult controls in available reference literature, suggesting that these cytokines might be involved in the local inflammatory process in these individuals. We found high variability in cytokine concentrations in the three biofluids and between JIA and controls. More studies are warranted to further explore the use of salivary, serum, and synovial fluid proteins as biomarkers in JIA.

## Electronic supplementary material

Below is the link to the electronic supplementary material.

Supplementary Material 1: Table S1. Cytokine concentrations in biofluids among children with JIA and TMJ arthritis and controls

## Data Availability

No datasets were generated or analysed during the current study.

## Abbreviations

JIA: Juvenile Idiopathic Arthritis
JIA-TMJ: Juvenile Idiopathic Arthritis – Temporomandibular Joint
NorJIA study: Norwegian Juvenile Idiopathic Arthritis Study
ILAR: International League of Associations for Rheumatology
DMARDs: Disease-Modifying Anti-Rheumatic Drugs
bDMARDs: Biologic DMARDs
MTX: Methotrexathe
TNFi: Tumor Necrosis Factor Alpha Inhibitor
PRgloVAS: Patient-Reported Global Assessment of Disease Impact on Overall Well-Being
PRpainVAS: Patient-Reported Pain VAS
MDgloVAS: Medical Doctor’s Global Assessment of Overall Disease Activity on a 10-cm VAS
TMJaw: The Temporomandibular Joint Juvenile Arthritis Working group
LOD: Level of Detection
LLOQ: Lower Level of Quantitation
GBI: Gingival Bleeding Index
OHI-S: Simplified Oral Hygiene Index
