# Supplementary material for: Cytokines in saliva, serum, and temporomandibular joint synovial fluid in children with juvenile idiopathic arthritis: An explorative cross-sectional study
Source: Pediatr Rheumatol Online J. 2025 Jun 17;23:66. doi: 10.1186/s12969-025-01118-y (PMC12172225; doi:10.1186/s12969-025-01118-y)
Supplement: Supplementary file 1 — Supplementary Material 1: Table S1. Cytokine concentrations in biofluids among children with JIA and TMJ arthritis and controls [file 12969_2025_1118_MOESM1_ESM.docx]

**Supplemental table 1. Cytokine concentrations in biofluids among children with JIA and TMJ arthritis and controls**

| **Cytokines** | **Saliva JIA-TMJ (n=13)**  **(pg/ml))**  **Median** **(IQR)** | **Saliva controls**  **(n=28)**  **(pg/ml)**  **Median** **(IQR)** | **Serum JIA-TMJ (n=11)**  **(pg/ml)**  **Median** **(IQR)** | **Serum controls (n=16)**  **(pg/ml)**  **Median** **(IQR)** | **TMJ-Synovial fluid (n=3) **** | | |
| --- | --- | --- | --- | --- | --- | --- | --- |
|  |  |  |  |  | **Patient 1**  **(pg/ml)** | **Patient 2**  **(pg/ml)** | **Patient 3**  **(pg/ml)** |
| IL-1b | 43.6 (21.0-116.1) | 21.9  (9.3-46.4) |  |  | 0.4 | 8.0 | 0.7 |
| IL-1ra | 23463.0  (12203.6- 42211.4) | 15201.2  (10366.4- 27364.0) |  |  | 41.4 | 6400 | 82.7 |
| IL-2 | 5.2 (2.5-8.5) |  |  |  | 1.4 | 31.5 | 2.9 |
| **IL-4** | 2.4 (1.9-3.4) | 1.6 (1.2-2.5) | 1.9 (1.4-3.2) | 1.6 (1.1-2.1) | 0.3 | 7.5 | 0.7 |
| IL-5 | 57.4 (10.4 – 83.9) | 26.4 (5.9-43.5) |  |  | 96.3 | 2270.0 | 116.6 |
| IL-6 | 3.9 (1.9 -5.8) | 2.7 (1.9-5.1) |  |  | 0.4 | 38.5 | 0.9 |
| IL-7 | 5.9 (5.9-25.5) | 5.9 (5.9-5.9) |  |  | 13.5 | 296.8 | 27.0 |
| **IL-8** | 334.7 (139.7-794.3) | 398.8 (202.6-643.7) | 3.4 (0.5-5.5) | 2.9 (0.5-4.0) | 5.2 | 23.0 | 2.1 |
| **IL-9** | 25.1 (17.2-36.2) | 15.2 (11.3-20.5) | 330.9 (318.7-352.1) | 320.3 (307.6-340.9) | 308.5 | 253.0 | 237.1 |
| IL-10 | 5.1 (2.7-7.9) | 0.6 (0.6-3.9) |  |  | 1.3 | 132.5 | 12.1 |
| IL-12 | 22.1 (7.1-25.8) | 0.8 (0.8-6.6) |  |  | 33.3 | 256.0 | 49.4 |
| **IL-13** | 1.2 (0.9-2.3) | 0.7 (0.5-1.2) | 0.9 (0.1-1.1) | 0.1 (0.1-1.7) | 2.1 | 23.0 | 3.2 |
| IL-15 | 39.1 (39.1-174.8) |  |  |  | 88.9 | 1956.5 | 177.9 |
| **IL-17** | 13.7 (10.2-21.9) | 7.4 (1.4-11.6) | 18.4 (16.4-24.7) | 17.7 (16.6-19.2) | 15.9 | 71.5 | 6.5 |
| **Eotaxin** | 2.6 (2.0-3.4) | 2.0 (1.7-2.5) | 38.8 (33.1-58.2) | 37.3 (30.2-46.1) | 27.8 | 13.5 | 25.2 |
| FGF basic | 19.1 (14.9-26.4) | 15.2 (12.3-18.4) |  |  | 4.7 | 104.0 | 9.5 |
| **G CSF** | 126.3 (82.0-173.7) | 100.0 (70.1-164.7) | 84.0 (62.3-113.4 | 66.7 (62.3-92.5) | 45.5 | 177.5 | 16.1 |
| GM CSF | 3.7 (2.5-6.6) | 2.3 (1.9-3.9) |  |  | 8.4 | 48.5 | 8.9 |
| IFNγ | 206.9 (176.4-232.7) | 188.1 (163.4-215.9) |  |  | 20.3 | 153.5 | 2.6 |
| **IP10** | 28.1 (16.7 -101.4) | 123.1 (65.6-240.7) | 182.4 (76.0-315.9) | 177.6 (133.6-223.7) | 39.9 | 40.0 | 127.5 |
| **MCP1 MCAF** | 25.7(14.7-51.9) | 18.4 (14.5-48.9) | 12.4 (9.1-22.4) | 10.6 (7.2-16.6) | 15.5 | 13.5 | 23.1 |
| **MIP1a** | 1.1 (0.7 -1.9) | 0.9 (0.7-1.3) | 0.9 (0.6-1.3) | 0.6 (0.4-1.0) | 0.6 | 2.0 | 0.2 |
| **PDGFbb** | 38.2 (24.3-48.0) | 13.7 (1.8-32.3) | 1340.2 (1090.6-2101.0) | 1249.5 (794.3-1565.4) | 397.2 | 890.0 | 55.9 |
| MIP1b | 3.6 (0.9-12.9) |  | 110.3 (102.6-114.7) | 108.7 (103.5-113.7) | 112.6 | 43.5 | 74.6 |
| **RANTES** | 22.4 (16.7-25.7) | 16.9 (13.5 -21.4) | 7108.5 (6325.6-7482.6) | 6375.3 (5984.6-6733.6) | 2158.0 | 100.0 | 1068.4 |
| **TNF** | 29.5 (21.9-68.7) | 22.7 (15.7-30.9) | 144.7 (131.3-150.8) | 144.7 (129.4-148.3) | 149.7 | 94.0 | 99.6 |
| VEGF | 144.3 (15.0-266.5) |  |  |  | 486.3 | 8536.5 | 607.2 |

** Three TMJ-synovial samples were excluded due to sample quality criteria and dilution factor above 0.98. **13 cytokines were detected in all media and groups (bold marking).** The following cytokines were excluded in both JIA-TMJ serum and controls-serum due to more than 60% of the samples were below the limit of detection: IL-1ra, IL-1b, IL-2, IL-5, IL-6, IL-7, IL-10, IL-12, IL-15, GM-CSF, IFNγ, FGF-basic, VEGF. The following cytokines were excluded in controls saliva due to more than 60% of the samples were below limit of detection: IL-2, IL-15, MIP1b, VEGF. One of the patients had two serum and three saliva samples and therefore the mean was calculated from those samples. JIA, juvenile idiopathic arthritis; TMJ, temporomandibular joint
